# Cognitive‐Behavioral Intervention for Children With Hematological Cancer Receiving Chemotherapy: A Randomized Controlled Trial

**DOI:** 10.1002/pon.70086

**Published:** 2025-01-19

**Authors:** Tenaw Gualu Melesse, William Ho Cheung Li, Janita Pak Chun Chau, Mulugeta Ayalew Yimer, Abdulkadir Mohamedsaid Gidey, Sewbesew Yitayih

**Affiliations:** ^1^ The Nethersole School of Nursing Faculty of Medicine The Chinese University of Hong Kong Hong Kong China; ^2^ Department of Pediatrics and Child Health Nursing College of Medicine and Health Sciences Debre Markos University Debre Markos Ethiopia; ^3^ Pediatric Hematology‐Oncology Unit Department of Pediatrics and Child Health School of Medicine College of Medicine and Health Sciences University of Gondar Gondar Ethiopia; ^4^ Pediatrics Hematology‐Oncology Division Department of Paediatrics and Child Health College of Health Sciences Addis Ababa University Addis Ababa Ethiopia; ^5^ Department of Psychiatry School of Medicine College of Medicine and Health Sciences University of Gondar Gondar Ethiopia

**Keywords:** cancer, chemotherapy, child, cognitive behavioral therapy, drug therapy, hematologic neoplasms, oncology, randomized controlled trial

## Abstract

**Objective:**

Cognitive‐behavioral intervention (CBI) has shown positive effects in improving psychological and health‐related outcomes in children with cancer. However, no evidence has been found in Ethiopia. This study aimed to evaluate the effects of CBI on anxiety, depression and quality of life (QoL) in Ethiopian children with hematological cancer receiving chemotherapy.

**Methods:**

A parallel, two‐armed, assessor‐blinded, randomized controlled trial was conducted among 76 children randomized (1:1) to receive CBI or usual medical care. The intervention group received five weekly face‐to‐face CBI sessions of 30–40 min each, which included an introduction to CBI; identifying and challenging maladaptive thoughts, beliefs and behavior; behavior activation; deep breathing exercises; and treatment evaluation and relapse prevention. The outcomes were measured at baseline (T0), immediately post‐intervention (T1) and 1 month post‐intervention (T2).

**Results:**

The intervention group showed a significant reduction in anxiety scores from T0 at T1 (*β* = −6.67, 95% CI [−9.16, −4.19], *p* < 0.001) and T2 (*β* = −8.14, 95% CI [−10.70, −5.57], *p* < 0.001), depression at T1 (*β* = −4.09, 95% CI [−6.94, −1.23], *p* = 0.005) and T2 (*β* = −6.12, 95% CI [−9.10, −3.13], *p* < 0.001) and improvement in QoL at T2 (*β* = 3.02, 95% CI [0.49, 5.56], *p* = 0.019) compared with the control group.

**Conclusions:**

CBI has positive effects in reducing anxiety and depression and in improving QoL in children with hematological cancer receiving chemotherapy. The results suggest the need to incorporate CBI into pediatric hematology‐oncology and studies on its long‐term effects and cost‐effectiveness are warranted.

**Trial Registration:** ClinicalTrials.gov (NCT05270655). Registered on 08 March 2022.

## 1 Background

Hematological cancer is the most common type of cancer in children under 19 years of age. It accounts for approximately 45% of the total childhood cancer worldwide [1, 2] and 50%–60% of the childhood cancer in Ethiopia [3, 4].

The shock of diagnosis; uncertainties about treatment outcomes, such as fear of death and relapse [5, 6]; treatment and chemotherapy side effects [7, 8]; changes in their physical body image; loss of functional mobility; and challenges related to transition to a new normal during treatment are highly challenging experiences for children [9, 10], especially during developmental transitions [11]. Nearly 90% of children undergoing cancer treatment experience at least one type of distress symptom [12], among which anxiety and depression are commonly reported [13, 14]. In a recent study, the pooled prevalence of anxiety and depression among children was found to be 13.9% and 20.4%, respectively [15].

Anxiety predicts fatigue [16], anticipatory pain, vomiting and nausea during subsequent medical procedures [17], thus affecting adherence, symptom management and treatment outcomes [10], and it increases off‐therapy distress symptoms [18]. Depression causes a loss of motivation to change behavior and may lead to nonadherence to treatment [10, 19], thus affecting prognosis and quality of life (QoL). Depression also increases adverse health outcomes, risk of relapse [20], the costs of treatment [21] and the mortality rate [22].

Nowadays, psychological interventions such as music‐based interventions [23], art therapies [24], play‐based interventions [25] and cognitive‐behavioral interventions (CBI) [26] have shown positive effects on reducing distress symptoms and health‐related outcomes in children with cancer.

CBI is the most recommended approach for its efficacy and enduring effects to manage distress symptoms in children with cancer [10, 27]. CBI comprises cognitive and behavioral techniques that help to modify distorted thoughts, negative feelings and dysfunctional behavior and to develop more adaptive thoughts, feelings and behavior [28]. It aims to directly involve patients in controlling their emotions and behavior and developing adaptive thoughts and behavior, and it emphasizes maintaining changes, preventing relapse and making the intervention sustainable [29].

A recent review has supported the positive effects of CBI on anxiety, depression and health‐related outcomes in children with cancer [26]. The interventions range from one to eight face‐to‐face CBI sessions of 30–60 min for each session, and the majority of the interventions are conducted one‐to‐one. However, most studies have had methodological limitations, such as the interventions not being guided by theoretical models, including heterogeneous participants and intervention approaches, and not reporting on follow‐up evaluations. Additionally, the studies have provided no evidence of the effects of the interventions in developing countries that have diverse cultural backgrounds such as Ethiopia.

Thus, the current study aimed to evaluate the effects of a theoretical model‐guided CBI on anxiety, depression and QoL in Ethiopian children with hematological cancer receiving chemotherapy.

## 2 Methods

### 2.1 Study Design

This study was a parallel, two‐armed, single‐blinded, repeated‐measure, randomized controlled trial (RCT). A well‐designed repeated‐measure RCT is the highest standard of intervention study design for establishing causal inference [30], and enables the measurement of changes in outcomes over time [31]. The study was prospectively registered with the ClinicalTrials.gov (NCT05270655) and reporting adhered to the CONSORT‐Outcomes 2022 extension [32].

### 2.2 Participants

Children aged ≥ 8 to < 18 years with hematological cancer, receiving chemotherapy and able to communicate in Amharic (grade 3 and above) and provide written parental consent and oral child assent were included. Children aged ≥ 8 years can easily participate in many CBI strategies [28], and provide reliable reports of their illness experiences [33]. Additionally, Ethiopian children gain substantial reading comprehension after grade 3, enabling them to complete self‐report questionnaires [34].

Children with known developmental, cognitive, intellectual or psychiatric problems; who were unable to collaborate during the study due to acute illness; or who were participating in another psychological or CBI based study or had previously participated in such a study were excluded.

### 2.3 Study Setting

The participants were recruited from the two pediatric hematology‐oncology wards and two outpatient departments of University of Gondar Specialized Hospital and Tikur Anbesa Specialized Hospital. The pediatric oncology units in the study hospitals have a total capacity of 77 beds and provide services for more than 900 new cases of cancer annually.

### 2.4 Interventions

#### 2.4.1 Intervention Group

The CBI was underpinned by Beck's cognitive model [35]; it was informed by the results of our systematic review [26], qualitative study (unpublished study), psychometric evaluation study [36], and pilot study (unpublished study) and by reviewing CBI guidelines [28, 37], and expert recommendations. The intervention group received five weekly one‐on‐one face‐to‐face CBI sessions of 30–40 min each. The CBI sessions took place in separate rooms located near pediatric oncology wards to provide privacy to participants and prevent information contamination. Each session was accompanied by home‐based practices in addition to the usual medical care. The intervention was delivered by a CBI trained nurse (in each hospital) with a master's degree and clinical experience.

The introduction to CBI comprised establishing a therapeutic relationship; introducing the intervention procedures; an initial assessment of the patient's problems; establishing a treatment plan; and introducing childhood cancer, such as causes, types and treatments. The parents attended the portion of the first session to help them understand the purpose of the study.

The second session comprised identifying, evaluating and challenging maladaptive thoughts, beliefs and behavior. Children were supported to identify, evaluate and challenge their distorted automatic thoughts and beliefs and replace them with functional thoughts and beliefs that acknowledged their personal strength and functionality.

The third session involved behavioral activation, with a focus on identifying and applying healthy coping behaviors and establishing behavior goals by identifying and introducing pleasant activities to increase activity, mode and function.

The fourth session consisted of deep breathing exercises to reduce stress and improve relaxation. This study adopted 4‐5‐6 breathing (i.e., breathing slowly through the nose for a count of four, holding the breath for a count of five and slowly exhaling the breath through the mouth for a count of six) and repeating the procedure three to four times.

The last session consisted of treatment evaluation and relapse prevention. It comprised a review of the intervention received, a discussion of maintenance of the use of CBI and relapse prevention strategies, receiving feedback and acknowledging the participant.

The participants were provided with worksheets containing a cognitive triad, a thought diary, an activity diary and the steps of the deep breathing exercises. Each session was conducted in a private room in each hospital through psychoeducation, questioning, guided discovery, discussion, drawing, writing and playing as appropriate the content.

#### 2.4.2 Control Group

The control group received the usual medical care. We used the usual care control, the commonly used control group type in our systematic review [26] and behavioral in psychotherapy research, to facilitate the comparison of the effectiveness of the intervention. To offer them attention and ensure adherence, the research assistants who did not know about the intervention group met them every week for 20–30 min to ask them about any concerns they may have had about the study. The research assistants contacted participants whose medical appointments were more than a week apart via phone call.

The participants in both groups were provided with an appointment slip. Additionally, a weekly telephone reminder was conducted 2 days before the appointment to ask the participants about any concerns they may have had about the study and to remind them about their next appointment.

### 2.5 Outcomes and Measurements

#### 2.5.1 Primary and Secondar Outcomes

The primary outcome, anxiety, and the secondary outcomes, depression and QoL, were measured before the intervention (T0, at baseline), immediately post‐intervention (T1, at 6 weeks) and 1 month post‐intervention (T2, at 10 weeks). Anxiety and depression were measured using the self‐report 25‐item Revised Child Anxiety and Depression Scale (RCADS‐25). The RCADS‐25 comprises a 15‐item anxiety scale and a 10‐item depression scale, and it is scored on a 4‐point Likert scale (0–3) [38]. It had strong validity and high reliability in the original study [39]. The scale has been translated into several languages for cross‐cultural use [38]. In this study, the Amharic version of the RCADS‐25 was validated and used, which demonstrated high validity and reliability (*α* = 0.95, 0.94 and 0.96 for the anxiety scale, depression scale and the total RCADS‐25, respectively) in an unpublished study. The total score is calculated by converting the row scores into T‐scores. A higher T‐score indicates higher anxiety and depression [38].

QoL was measured using the self‐report Pediatric Quality of Life Inventory 4.0 Generic Core Scale (PedsQL 4.0 GCS). It comprises 23 items (physical functioning = 8 items, emotional functioning = 5 items, social functioning = 5 items and school functioning = 5 items). It is scored on a 5‐point Likert scale (0–4) [40, 41]. It showed strong validity and acceptable reliability in the original study [40]. It has been validated in several languages. This study used the Amharic version of PedsQL 4.0 GCS for the age groups of 8–12 and 13–18 years, which had high validity and strong reliability (*α* = 0.96) [36]. The total score is calculated by transforming the 0–4 scale items to a 0–100 scale. A higher score indicates higher QoL [41].

#### 2.5.2 Other Outcomes

At T0, the participants completed the self‐report sociodemographic datasheet, and the clinical data were collected from their medical records.

A self‐administered satisfaction questionnaire was developed with a general focus on the patient's overall satisfaction with the CBI by reviewing the literature [42]. It was administered to the participants in the intervention group at T1. It comprises five items, with each item rated on a 5‐point Likert scale (0–4). The total score ranges from 0 to 20, and the high score indicates the high acceptability of the intervention.

### 2.6 Sample Size Determination

The sample size was calculated based on the effect size of CBI on the primary outcome, anxiety, from previous studies included in our review, which had Cohen's *d* values ranging from 0.69 to 3.93 [26]. Considering the smallest effect of 0.69 and using G*Power (version 3.1.9.7), a sample size of 34 participants per group would give the study 80% power to detect an effect size of at least 0.69 between the study groups at a 5% significance level (two‐sided) using the independent sample *t*‐test. Estimating a 15% attrition rate, a total of 80 participants were targeted for recruitment.

### 2.7 Randomization

As the study included participants with a wide age range (≥ 8 to < 18 years old), we stratified the participants according to their age groups to balance the comparability of the study groups. The participants were stratified based on their age groups that is, school age (≤ 12 years) and adolescents (≥ 13 years) [43]. The participants in each stratum at each hospital were then randomized in a 1:1 allocation using a permuted block randomization with random mixed block sizes of 2 and 4. An independent researcher (in each hospital) generated the random sequence using a computer‐generated random list. Then the participants were allocated to the study groups in each hospital according to their sequence of enrollment in the study and the corresponding group identifiers in each block in the random group sequence.

### 2.8 Allocation Sequence

The group label of the participants was placed in sequentially numbered sealed opaque envelopes. The allocation sequence was kept only by an independent researcher in each hospital and concealed until the treatment assignment. After the baseline measurements, the envelopes were opened by the intervention providers in front of the participants and parents.

### 2.9 Blinding

The outcome assessors (nurses) were blinded to the study groups and study objectives. Due to the nature of the intervention, it was not possible to blind the participants or intervention providers. To ensure the blinding of the outcome assessors, the study participants were advised not to disclose their group assignment to the outcome assessors.

### 2.10 Data Analysis

The data were analyzed using IBM SPSS 26. Participant characteristics and outcome variables were summarized using descriptive statistics. The normality of the continuous variables was assessed using the skewness and kurtosis values and the Shapiro–Wilk test. For a sample size of 50–300, an absolute z‐score of less than 3.29 for the skewness and kurtosis values is considered to indicate a normal distribution [44].

The homogeneity of the participant characteristics and outcome variables between the study groups was compared using an independent *t*‐test, chi‐square test and Fisher's exact test as appropriate at the baseline. The participants were analyzed in the group to which they were originally assigned [45]. As the data collected from the same subject in repeated measurements across time are likely to be correlated and violate the assumptions of independent observations [31], the generalized estimation equations model was adopted to accommodate the correlations of repeated observations and normal and non‐normal outcome data and to examine the changes in the outcomes across the time points. The significance level was set at 5% (two‐sided).

### 2.11 Ethics Approval

This study was performed in line with the principles of the Declaration of Helsinki. Informed written parental consent and oral child assent were obtained. Ethical approval was obtained from the Joint Chinese University of Hong Kong‐New Territories East Cluster Clinical Research Ethics Committee (CREC: 2021.729‐T), the Institutional Ethical Review Board of the University of Gondar (VP/RTT/05/8/22), and the Ethics and Research Committee of the Department of Pediatrics and Child Health, Addis Ababa University (Ped/Mf/343/14).

## 3 Results

### 3.1 Participant Recruitment

**FIGURE 1:**
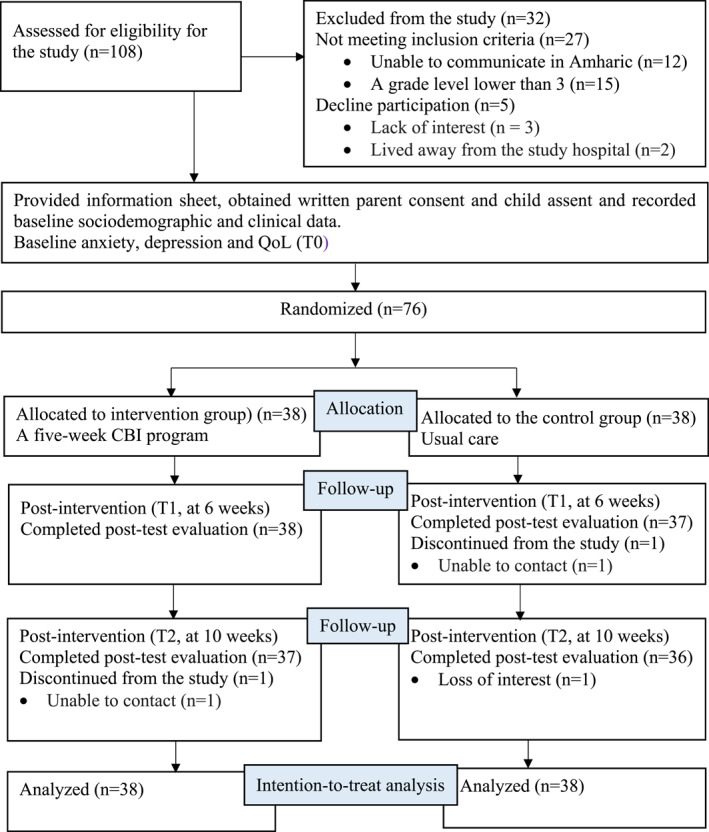
CONSORT diagram.

From April to October 2022, a total of 108 potential participants were assessed for study eligibility. The last data collection was conducted on October 20, 2022, as no further potential participants were identified. Twenty‐seven patients did not meet the eligibility criteria and five participants declined to join the study. Finally, 76 children provided written parental consent and they were randomly assigned in a 1:1 allocation to the intervention and control group. The recruitment, participation and overall attrition rates were 70.4%, 93.8% and 3.9%, respectively (Figure 1).

### 3.2 Characteristics of the Participants

Table 1 shows the baseline characteristics of the participants in the study groups, which were comparable.

**TABLE 1:** Sociodemographic and clinical characteristics of the total sample and comparison of the intervention and control groups.

		Mean ± SD or *n* (%)				
Participant characteristics		Total sample (*N* = 76)	CBI group (*n* = 38)	Control group (*n* = 38)	*t* or *χ*2(df)	*p*‐value
Age (year)	Range = 9–17 years	12.74 ± 1.87	12.82 ± 1.87	12.66 ± 1.88	0.367	0.715^a^
Gender	Male	41 (53.9)	22 (57.9)	19 (50.0)	0.477 (1)	0.490^b^
	Female	35 (46.1)	16 (42.1)	19 (50.0)		
Educational level	Primary school	65 (85.5.)	33 (86.8)	32 (12.2)	0.106 (1)	0.744^b^
	Secondary school	11 (14.5)	5 (13.2)	6 (15.8)		
Religion	Orthodox christian	49 (64.5)	26 (68.4)	23 (60.5)	0.525 (2)	0.769^b^
	Muslim	16 (21.1)	7 (18.4)	9 (23.7)		
	Protestant	11 (14.5)	5 (13.2)	6 (15.7)		
Residential address	Rural	50 (65.8)	23 (60.5)	27 (71.1)	0.935 (1)	0.333^b^
	Town	26 (34.2)	15 (39.5)	11 (28.9)		
Primary caregiver's educational level	No formal education	34 (44.7)	16 (42.1)	18 (47.4)	NA	0.923^c^
	Primary school	20 (26.3)	10 (26.3)	10 (26.3)		
	Secondary	13 (17.1)	7 (18.4)	6 (15.8)		
	College and above	9 (11.8)	5 (13.2)	4 (10.5)		
Household size	< 5	41 (53.9)	22 (57.9)	19 (50.0)	0.477 (1)	0.490^b^
	≥ 5	35 (46.1)	16 (42.1)	19 (50.0)		
Family monthly	< 2500 (< 48.1)	33 (43.4)	17 (44.7)	16 (42.1)	0.654 (2)	0.721^b^
Income (ETB	≥ 2500–5000 (≥ 48.1–96.2)	31 (40.8)	14 (36.8)	17 (44.7)		
/USD)	≥5000–10000 (≥96.2–192.3)	12 (15.8)	7 (18.4)	5 (13.2)		
Enrolled in CBHI	Yes	45 (59.2)	20 (52.6)	25 (65.8)	1.362 (1)	0.243^b^
	No	31 (40.8)	18 (47.4)	13 (34.2)		
Diagnosis	ALL	41 (53.9)	20 (52.6)	21 (55.3)	NA	0.999^c^
	NHL	17 (22.4)	9 (23.7)	8 (21.1)		
	HL	13 (17.1)	6 (15.8)	7 (18.4)		
	AML	5 (6.6)	3 (7.9)	2 (5.3)		
Treatment modality	Chemotherapy alone	68 (89.5)	35 (92.1)	33 (86.8)	NA	0.711^c^
	Chemotherapy and radiation	8 (10.5)	3 (7.9)	5 (13.2)		
Duration of treatment	< 6	33 (43.4)	18 (47.4)	15 (39.5)	0.664 (2)	0.717^b^
	≥ 6–12 months	23 (30.3)	10 (26.3)	13 (34.2)		
	≥ 12 months	20 (26.3)	10 (26.3)	10 (26.3)		
Comorbidities	Yes	0 (0.0)	0 (0.0)	0 (0.0)	NA	NA
	No	76 (100)	38 (100)	38 (100)		

*Note:* 1 USD ∼ 52 ETB (Bank exchange rate during the data collection time).

Abbreviations: CBHI = community‐based health insurance; ETB = Ethiopian birr; NA = Not applicable; USD = United States dollars; *N* = total number of participants; *n* = number of participants per group; *t* = independent *t*‐test; df = degree of freedom; *t* = t‐statistics; *χ* ^2^ = Chi‐square test.

^a^ independent *t*‐test.

^b^ Chi‐square test.

^c^ Fisher's exact tests.

### 3.3 Baseline Outcome Variables

All of the outcome variables were normally distributed (the z‐scores of the skewness and kurtosis ranged from −0.82 to 1.24 and −1.56 to −0.59, respectively, with *p* > 0.05 in the Shapiro–Wilk test). There were no significant baseline outcome differences between the study groups for all of the outcome variables (Table 2).

**TABLE 2:** Baseline outcome variables of the total sample and comparison of the intervention and control groups.

	Mean ± SD				
	Total sample (*N* = 76)	Intervention group (*n* = 38)	Control group (*n* = 38)	t	*p*‐value
Anxiety	58.39 ± 7.42	59.87 ± 7.05	58.89 ± 7.83	0.570	0.571
Depression	57.87 ± 7.63	57.45 ± 8.33	58.29 ± 6.94	−0.479	0.634
PedsQL™ 4.0 GCS (overall score)	45.95 ± 7.97	45.62 ± 7.87	46.28 ± 8.16	−0.356	0.723

Abbrevaitions: *N* = total number of participants; *n* = number of participants per group; *t* = independent *t*‐test.

### 3.4 Completers and Non‐completers of the Study

The results of an independent *t*‐test and Fisher's exact test showed no significant differences for all characteristics and baseline outcome variables between the completers and non‐completers.

### 3.5 Effects of the CBI on Outcomes

Given that no covariates were identified in the baseline measurements, a crude GEE model was adopted and the results are presented below.

#### 3.5.1 Effects of the CBI on Anxiety

There were no significant differences in the mean anxiety score between the study groups at the baseline (*β* = 0.97, 95%CI [2.33, 4.28], *p* = 0.564). In the control group, the mean score of anxiety was reduced by *β* = −1.04, 95% CI [−2.84, 0.77], *p* = 0.260 at T1 and *β* = −1.83, 95% CI [−3.86, 0.20], *p* = 0.078 at T2 in comparison with the baseline score. At T1 and T2, the change in the mean score of anxiety in the intervention group showed a significant decrement (*β* = −6.67, 95% CI [−9.16, −4.19], *p* < 0.001 and *β* = −8.14, 95% CI [−10.70, −5.57], *p* < 0.001, respectively) in addition to the changes in the control group at the corresponding time points. The effect sizes of CBI on anxiety (Cohen's *d* = 0.58 and 0.82 at T1 and T2, respectively).

#### 3.5.2 Effects of the CBI on Depression

At the baseline, there were no significant differences in the mean depression score between the study groups (*β* = −0.84, 95%CI [−4.24, 2.56], *p* = 0.628). The control group showed a small reduction in the mean score of depression at T1 (*β* = −0.55, 95% CI [−3.07, 1.98], *p* = 0.671) and T2 (*β* = −1.12, 95% CI [−3.69, 1.45], *p* = 0.392) compared with the baseline mean score. In the intervention group, the changes in the mean score of depression showed a significant decrement (*β* = −4.09, 95% CI [−6.94, −1.23], *p* = 0.005 and *β* = −6.12, 95% CI [−9.10, −3.13], *p* < 0.001 at T1 and T2, respectively) on top of the changes in the control group at the corresponding time points. The effect sizes of CBI on depression (Cohen's *d* = 0.59 and 0.86 at T1 and T2, respectively).

#### 3.5.3 Effects of the CBI on QoL

At the baseline, the mean QoL score between the study groups was not significantly different (*β* = −0.66, 95% [−4.21, 2.90], *p* = 0.718). The control group showed a small increment in the mean QoL score (*β* = 0.29, 95% CI [−0.88, 1.47], *p* = 0.619 and *β* = 0.43, 95% CI [−0.92, 1.78], *p* = 0.534 at T1 and T2, respectively) compared with the baseline mean QoL score. The changes in the mean QoL score in the intervention group showed a non‐significant improvement at T1 (*β* = 1.91, 95% CI [−0.51, 4.33], *p* = 0.122) and a significant improvement at T2 (*β* = 3.02, 95% CI [0.49, 5.56], *p* = 0.019) in addition to the changes in the control group at the corresponding time points. The effect sizes of CBI on QoL (Cohen's *d* = 0.20 and 0.40 at T1 and T2, respectively).

**FIGURE 2:**
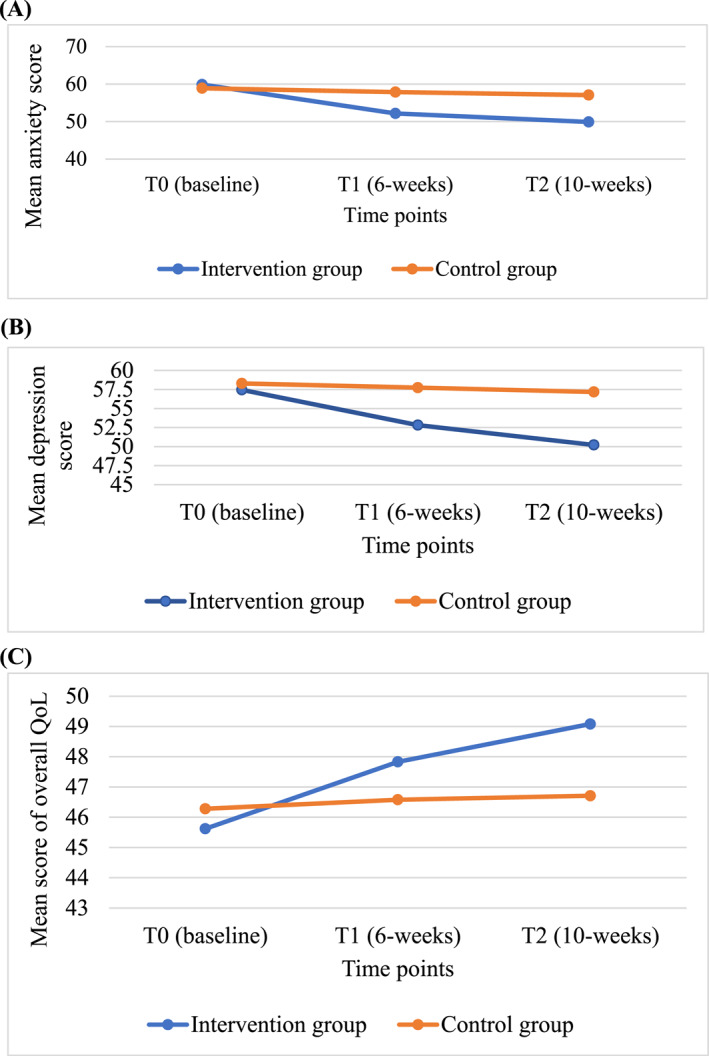
Changes in the anxiety (A), depression (B) and QoL (C) across time between the intervention and control groups.

The changes in the anxiety, depression and QoL mean scores across time are shown in Figure 2.

#### 3.5.4 Acceptability of the CBI

All 37 participants who completed the satisfaction questionnaire were satisfied or very satisfied with the CBI. Nineteen (51.4%), sixteen (43.2%) and two (5.4%) of the participants reported that the CBI helped them manage much, a great deal and to some extent of their health problems, respectively. All the participants reported that they intended to often or always use CBI and suggested that the program was important or very important for other children with cancer. In addition, all of the participants indicated that the intervention strategies were easy or very easy to practice. The results showed the high satisfaction of the participants and the acceptability of the intervention. In addition, no adverse events directly related to the intervention were reported and thus, the program was proven safe in this study.

## 4 Discussion

To the best of our knowledge, this study is the first study to evaluate the effects of theory‐driven CBI on distress symptoms and QoL of children with cancer in Ethiopia. The high satisfaction of the participants with the intervention and the high study completion rate suggest the program's acceptability and thus, this helps to experience the program's intended benefits and provides an important insight into making CBI widely applicable.

Regarding the program effect, the findings show that CBI significantly reduces anxiety and depression in the intervention group immediately post‐intervention and this effect remains significant at 1‐month follow‐up, while the control group shows no significant change from the baseline. The results are congruent with the findings of previous studies [46, 47, 48, 49]. The positive effects of CBI on anxiety and depression might be because it helps with understanding distorted thoughts, feelings and behavior and developing adaptive cognition and coping behavior to alleviate stressful situations [29, 50]. However, previous studies have evaluated different CBI formats and the comparative effectiveness of the different forms of CBI warrants further study. On the other hand, although the participants in the intervention group experience improvements in the QoL across time points, the intervention shows a significant effect only at 1‐month post‐intervention. The improvements in QoL might be due to developing positive thoughts and coping behaviors, that reduce distress symptoms [51]. However, the findings contrast with a previous study that showed the positive effect of CBI after the intervention [52]. In this study, the non‐significant effect of CBI immediately post‐intervention could be related to the short duration of the intervention and the relative stability of QoL. Generally, studies on the long‐term effects of CBI on anxiety, depression and QoL in children with cancer are limited, and further studies are warranted.

Most CBI interventions are psychologist‐administered in previous studies [46, 47, 48, 49]. Our study can be considered the first for evaluating nurse‐administered CBI for children with cancer and produces similar positive outcomes to the previous studies. As nurses are primary caretakers of patients, Nurse‐administered CBI might have high adoption and sustainability in clinical practice. They could also have the potential to adapt to delivery via different methods such as telehealth, but its effects require further studies. However, this study included a non‐intervention control group, and we couldn't compare the effectiveness of the CBI with other interventions that could require limited resources to integrate into clinical practice.

### 4.1 Limitations of the Study

Some limitations of the study should be acknowledged when interpreting the findings. First, although there might have been several factors moderating the changes in the outcomes in response to CBI, this study did not evaluate the moderators of the outcomes. Second, there was a potential risk of contamination when some patients came in contact with each other during their in‐patient stay. Third, conclusions on the long‐term effects of the intervention could not be certainly drawn. Fourth, this study used a non‐intervention control group, and thus, we couldn't compare the effectiveness of the CBI with other interventions in the control group. Furthermore, it may be worth exploring participants' perceptions and experiences with CBI using qualitative interviews.

### 4.2 Clinical and Research Implications

The findings demonstrated that CBI significantly improves anxiety, depression and QoL. The positive effects 1 month post‐intervention suggested that CBI may help with maintaining positive changes and preventing relapse. Therefore, the results support the need to integrate CBI strategies into pediatric cancer care practice. Additionally, CBI trained nurses are probably the most suitable professionals to adopt and sustain CBI because they are primarily responsible for caring for patients and have frequent contact with patients. However, solid knowledge of the cognitive model and its essential components and mechanism of action is essential in administering the intervention. Intensive training is required to ascertain an intervener's competence to deliver the intervention. Additionally, some degree of flexibility may be required based on individual differences in administering CBI, such as allowing the presence of parents with limited roles or varying the length of sessions. Furthermore, training clinicians may require more resources, and thus the integration of CBI into clinical practice requires the commitment of hospital managers, clinicians and other stakeholders.

Studies that evaluate moderators and mediators are important to optimize who the CBI program is delivered to and understand how the program works. Studies that use cluster randomization are suggested to reduce information contamination between the study groups. Additionally, studies evaluating CBI with an active comparison group, the long‐term effects and cost‐effectiveness of the CBI are strongly recommended. Future studies that use qualitative process data can help explore the participants' experiences of the intervention to further improve the effectiveness of the intervention.

## 5 Conclusion

CBI has positive effects on reducing anxiety and depression and improving QoL in children with hematological cancer. The results suggest the need to incorporate CBI into pediatric hematology‐oncology practice. Further well‐designed studies on the long‐term effects of CBI and its cost‐effectiveness are warranted.

## Author Contributions

Conceptualization, methodology, analysis, writing‐original draft of the manuscript [Tenaw Gualu Melesse], methodology, analysis, writing ‐ reviewing and editing, and supervision [William Ho Cheung Li], conceptualization, methodology, writing ‐ reviewing and editing, and supervision [Janita Pak Chun Chau], and project administration and management [Mulugeta Ayalew Yimer, Abdulkadir Mohamedsaid Gidey and Sewbesew Yitayih]. All authors read and approved the final manuscript.

## Consent

Informed written parent consent and oral child assent were obtained from all participants.

## Conflicts of Interest

The authors declare that they have no financial or non‐financial interests to declare.

## Data Availability

The data that support the findings of this study can be obtained from the primary author upon reasonable request.
